# Self-Compassion Facets as Mediators of the Relationship Between Self-Esteem and Well-Being: A Path Analysis Approach

**DOI:** 10.5964/ejop.17201

**Published:** 2025-11-28

**Authors:** Jaime Navarrete, Corel Mateo-Canedo, Adrián Pérez-Aranda, Selena Russo, Stefano Ardenghi, Federico Zorzi, Giulia Rampoldi, Maria Grazia Strepparava, Jesús Montero-Marín, Marco Bani

**Affiliations:** 1 Teaching, Research & Innovation Unit, Parc Sanitari Sant Joan de Déu, Sant Boi de Llobregat, Spain; 2 CIBER of Epidemiology and Public Health (CIBERESP), Madrid, Spain; 3 Department of Basic Psychology, Universitat Autònoma de Barcelona, Cerdanyola del Vallès, Spain; 4 Department of Clinical and Health Psychology, Universitat Autònoma de Barcelona, Cerdanyola del Vallès, Spain; 5 School of Medicine and Surgery, University of Milano-Bicocca, Monza, Italy; 6 Fondazione IRCCS San Gerardo dei Tintori, Monza, Italy; 7 Department of Pedagogy, Psychology, and Philosophy, University of Cagliari, Cagliari, Italy; 8 Department of Psychiatry, University of Oxford, Warneford Hospital, Oxford, United Kingdom; Dublin City University, Dublin, Ireland

**Keywords:** self-compassion, Sussex-Oxford Compassion Scales, self-esteem, well-being, path analysis, psychometric validation

## Abstract

Self-esteem and self-compassion are both associated with psychological well-being, but they differ in their underlying mechanisms. Some studies have found that self-compassion mediates the relationship between self-esteem and well-being, yet they have overlooked the distinct roles of its cognitive, affective, and behavioral components. This study examined whether specific self-compassion facets mediate the relationship between self-esteem and well-being, using the Italian adaptation of the Sussex-Oxford Compassion Scales (SOCS), whose psychometric properties were also assessed. A cross-sectional survey was conducted among 408 Italian high school and undergraduate healthcare students. Participants completed the SOCS, the Rosenberg Self-Esteem Scale, and the Short Warwick-Edinburgh Mental Well-being Scale. The Italian SOCS was psychometrically evaluated through confirmatory factor analyses and showed good validity and reliability. Correlations and path analyses were used to examine the mediating roles of cognitive, affective, and behavioral self-compassion facets. Emotional and behavioral components of self-compassion — such as “feeling for one’s suffering”, “tolerating uncomfortable feelings”, and “acting to alleviate suffering” — significantly mediated the link between self-esteem and well-being. These facets accounted for 39% of its variance, while cognitive components did not show significant effects. These findings highlight the importance of affective and behavioral self-compassion in therapeutic interventions, emphasizing their role in enhancing well-being.

Self-esteem, as defined by Rosenberg (1965), is an overall assessment of one’s worth as a person and plays a vital role in mental health and quality of life. High levels of self-esteem are often associated with numerous positive psychological outcomes, such as increased happiness, resilience, and overall well-being (Orth & Robins, 2014; Sowislo & Orth, 2013). This highlights the importance of self-esteem in fostering a healthy self-concept and promoting adaptive behaviors, making it a significant target of psychotherapeutic interventions. However, programs addressed at enhancing self-esteem have generally produced modest effects (Muris & Otgaar, 2023), which might be explained by the observation that self-esteem is often highly resistant to change (Neff, 2003b) and tends to be experienced more as a result of perceiving oneself as successful in comparison to others rather than a precursor to well-being (Neff, 2011).

Self-compassion, on its part, has been defined in various ways; Strauss et al. (2016) conducted an exhaustive work of revising different definitions and concluded that self-compassion is the composite of five facets:

- Recognizing suffering.
- Understanding that suffering is a part of every human experience.
- Feeling empathy and relating to people who are suffering.
- Tolerating unpleasant feelings.
- Taking action to ease discomfort.

Self-compassion has received increased attention from researchers in recent decades, as it has been considered a pivotal aspect of the so-called “third wave” of cognitive-behavioral therapy (CBT), alongside mindfulness, acceptance, and spirituality (Jahoda et al., 2017). Research suggests that self-compassion is associated with lower levels of anxiety and depression, as well as higher levels of life satisfaction, positive affect, and optimism (Muris & Petrocchi, 2017; Zessin et al., 2015). On top of that, self-compassion can be successfully trained through “third wave” CBT programs (Ferrari et al., 2019; Wilson et al., 2019), which have also shown positive effects on well-being in different populations (Millard et al., 2023).

It seems clear that, although closely related, self-esteem and self-compassion refer to different aspects. In a recent review on this topic, Muris and Otgaar (2023) concluded that while the correlation between both constructs is large, some key differences exist:

- Self-esteem implies comparison to others, whereas self-compassion does not.
- Self-esteem seems to be associated with the activation of the sympathetic threat system, while self-compassion would be linked to the parasympathetic soothing system.
- Unlike self-compassion, self-esteem is a cognitive appraisal process that lacks an inherent coping mechanism.

This last point is particularly relevant, since it could be a potential reason behind the fact that interventions aimed at promoting self-compassion have shown better outcomes than those aimed at enhancing self-esteem (Muris & Otgaar, 2023; Neff, 2011).

Cultivating self-compassion implies, to some degree, training particular abilities to deal with suffering — abilities that span cognitive, emotional, and behavioral domains (Gilbert, 2009; Halamová & Kanovský, 2021; Kanov et al., 2004; Tiwari et al., 2020). “Recognizing suffering” and “understanding the universality of suffering” (cognitive facets) entail a rational process that encourages balanced judgments and helps individuals perceive their distress as part of a shared human experience rather than as something exceptional. On the other hand, the affective facets — “feeling for the person’s suffering” and “tolerating uncomfortable feelings” — require emotional openness and engagement with distress, rather than avoidance. Finally, the behavioral facet, “acting to alleviate suffering”, involves a proactive orientation toward addressing and potentially transforming the situation. Thus, self-compassion encompasses not only rational acknowledgment of suffering but also emotional and behavioral responses that may strengthen coping strategies. These latter facets, in particular, could represent valuable therapeutic targets due to their potential to enhance psychological well-being.

As mentioned earlier, well-being is closely related to both self-compassion and self-esteem, and previous research has explored the interplay among these three constructs. Given that self-esteem precedes self-compassion (Donald et al., 2018), some studies have examined the mediating role that this latter plays in the interaction between self-esteem and different aspects related to mental well-being: Bugay-Sökmez et al. (2023) observed that the relationships between self-esteem, brooding, and co-rumination were mediated by self-compassion; Holas et al. (2023) reported a similar finding regarding social anxiety; and self-compassion was found to be a moderator of the influence of self-esteem on mental health (Marshall et al., 2015). However, these studies used the Self-Compassion Scale (SCS; Neff, 2003a) which, although widely used, does not clearly differentiate between the cognitive, affective, and behavioral components of self-compassion. Neff’s model (Neff, 2003b) does differentiate between positive (“self-kindness”, “common humanity”, and “mindfulness”) and negative (“self-judgement”, “isolation”, and “over-identification”) facets, and two studies included this distinction in mediation analyses observing that positive facets mediated the relationship between self-esteem and well-being (Pandey et al., 2021), and that both — positive and negative — mediated the link between self-esteem and self-forgiveness (Pandey et al., 2023). Although these distinctions between positive and negative facets of self-compassion can be found in various studies, there is no agreement on how the SCS should be used in this regard (Strauss et al., 2016), and this measure has been charged with unsatisfactory psychometric properties in some cases (Pandey et al., 2021; Pfattheicher et al., 2017), which suggests that exploring new models of self-compassion might be useful.

The present study aims to enhance the understanding of the relationship between self-esteem, self-compassion, and well-being by exploring the mediating role of specific self-compassion facets (cognitive, affective, and behavioral) in the relationship between self-esteem and well-being. We utilized the Sussex-Oxford Compassion Scales (SOCS; Gu et al., 2020), a validated instrument based on the model proposed by Strauss et al. (2016). As a preliminary step to this analysis, we adapted the SOCS to Italian; the psychometric properties of the Italian version of the SOCS are also presented in this work.

## Method

### Design and Participants

This is a cross-sectional study in which a convenience sample of Italian high-school students interested in healthcare programs and undergraduate healthcare students at a north Italian university were invited to participate. Participation was encouraged with the chance of winning a €50 gift card, randomly awarded to one participant who completed the survey. Inclusion criteria were: (a) being 18 or older, (b) being native Italian speaker, and (c) signing the informed consent to participate. No exclusion criteria were applied.

### Procedure

Undergraduate healthcare students received an email invitation from the principal investigator, describing the study and providing the link to the survey, which required approximately 20 minutes. High school students aged ≥ 18 were invited to participate in three Open Days showcasing healthcare courses, which took place from December 2022 to March 2023.

This study was approved by the Ethics Committee of the University of Milano-Bicocca (No. 707, Protocol 0066628/2022) and complied with the Declaration of Helsinki. The SOCS adaptation to the Italian context followed standard forward and reverse translation procedures. Initially, three Italian clinical psychologists with good English proficiency provided a conceptual translation of the questionnaire items, discussing discrepancies and reaching a consensus on the item formulation. Then, an independent bilingual translator back-translated the resulting Italian version. Finally, an English native reviewed the two English versions of the SOCS to identify eventual incongruency in phrasing.

### Instruments

#### Sociodemographic Characteristics

Information about gender, age, studies, employment status, and income was collected.

#### Sussex-Oxford Compassion Scales

This questionnaire (SOCS; Gu et al., 2020) consists of two scales: one measuring self-compassion (SOCS-S) and the other measuring compassion for others (SOCS-O). Each includes 20 items that assess compassion through five dimensions: recognizing suffering, understanding the universality of suffering, feeling empathy for the person suffering, tolerating uncomfortable feelings aroused in response to suffering, and being motivated to act to alleviate suffering. Respondents rated items on a Likert scale (1 = *Not true at all*, 5 = *Always true*), and a total score was obtained by summing the responses, with higher scores indicating higher levels of compassion (Range 20–100). Authorization from the original authors was obtained for translating/validating the Italian version of the SOCS, and a detailed explanation of its psychometric properties can be found in the Results section. The Italian version of both scales can be found in the Supplementary Materials.

#### Rosenberg Self-Esteem Scale

This 10-item questionnaire (RSE; Rosenberg, 1965) evaluates perceived self-worth by assessing both positive and negative feelings about the self. Respondents rated items on a Likert scale (0 = *Strongly disagree*, 4 = *Strongly agree*), and a total score was calculated by summing the responses (Range 0–40), with higher scores indicating higher levels of self-esteem. The Italian adaptation of the RSE (Prezza et al., 1997) was used, showing high internal consistency in our sample (ɑ = 0.85).

#### Short Warwick-Edinburgh Mental Well-Being Scale

This 7-item questionnaire (SWEMWBS; Stewart-Brown et al., 2009) evaluates well-being through items that capture positive feelings and functioning, such as feeling optimistic, relaxed, and useful. Respondents rated items on a Likert scale (1 = *None of the time*, 5 = *All of the time*), and a total score was obtained by summing the responses. Higher scores indicate higher levels of mental well-being (Range 7–35). The Italian adaptation of the SWEMWBS (Gremigni & Stewart-Brown, 2011) was employed, demonstrating very good internal consistency in our sample (ɑ = 0.82).

#### Measures for Convergent Validity of the Italian Version of the SOCS

##### Self-Compassion Scale-Short Form

Developed as an abbreviated version of the original scale (Neff, 2003a), this questionnaire (SCS-SF; Raes et al., 2011) was designed to measure an individual’s level of self-compassion. It consists of 12 items that reflect the three main components of self-compassion according to Neff’s model: self-kindness versus self-judgement, common humanity versus isolation, and mindfulness versus over-identification. Respondents rated items on a Likert scale (1 = *Almost never*, 5 = *Almost always*), and a total score was obtained by calculating the average of all the items. The Italian adaptation of the SCS-SF (Petrocchi et al., 2014) was used, and it presented very good internal consistency in our sample (ɑ = 0.86).

##### Brief Interpersonal Reactivity Index

This 16-item scale (B-IRI; Ingoglia et al., 2016) is a short version of the original 28-item questionnaire (Davis, 1980) and evaluates empathy through four distinct subscales: “perspective taking”, “fantasy”, “empathic concern”, and “personal distress”. In our study, however, the “fantasy” subscale was not included due to its controversial use in literature, resulting in a 12-item questionnaire. This decision was based on ongoing conceptual and empirical concerns regarding the construct validity of the fantasy dimension. As noted by Nomura and Akai (2012), the fantasy subscale evaluates respondents’ tendency to become imaginatively involved with fictional characters and scenarios, a construct that may not reflect empathy in the same way as the other subscales, which focus on interpersonal contexts involving real people. Respondents rated items on a Likert scale (1 = *Does not describe me well*, 5 = *Describes me very well*). Subscale scores were calculated by averaging the responses for each dimension, with higher scores indicating higher levels of that specific aspect of empathy. The Italian version of the B-IRI (Ingoglia et al., 2016) was utilized, and its internal consistency in our sample was good for the first two subscales (ɑ = .72, .81) and acceptable for “personal distress” (ɑ = .65).

##### Fear of Compassion Scales

This questionnaire (FCS; Gilbert et al., 2011) was designed to assess individuals’ apprehension and discomfort about expressing compassion towards others (FCS-O), receiving compassion from others, and expressing compassion towards oneself (FCS-S). In the present study, the subscale assessing fear of receiving compassion from others was excluded, as it was not aligned with either the study’s objectives or the hypotheses concerning construct validity. The adaptation used for this study consisted of 17 items (10 items for FCS-O and 7 for FCS-S) that respondents rated on a Likert scale (0 = *Never*, 4 = *Always*). A total score for each scale was derived by calculating the average of the responses, with higher scores indicating greater fear of compassion. The Italian adaptation of the FCS (Dentale et al., 2017) was employed, demonstrating strong internal consistency in our sample (ɑ = .82 for FCS-O and ɑ = .84 for FCS-S).

##### Burnout Clinical Subtype Questionnaire-12 Item

This questionnaire (BCSQ-12; Montero-Marin et al., 2011), a short version of the original 36-item questionnaire (Montero-Marín & García-Campayo, 2010), was designed to assess burnout across three dimensions: “overload”, “lack of development”, and “neglect”. Participants rated each item on a Likert-type scale with seven response options (1 = *Completely disagree*, 7 = *Completely agree*). A score for each subscale was calculated by averaging the responses, with higher scores indicating higher levels of burnout. Permission from the original authors was obtained for translating/validating the Italian version of the BCSQ-12. The psychometric properties of the scale were very good, with high internal consistency in its three subscales (ɑ = 0.87, .90, and .86, respectively).

### Data Analysis

Data analysis was performed using SPSS v29 and Mplus v7.4. All variables were descriptively analyzed (mean, standard deviation, range, skewness, kurtosis, frequency, and percentages).

As a previous step to conducting the primary analysis of the present study, the factor structures of the Italian versions of the SOCS-O and SOCS-S were assessed. Confirmatory factor analyses (CFAs) with a diagonally weighted least squares (WLSMV) estimation method were conducted to assess dimensionality. Sample size adequacy for the CFA was evaluated based on a commonly accepted rule of thumb recommending a ratio of 10 participants for each item in the scale (Kline, 1994). The models proposed by Gu et al. (2020) were tested for both scales: all 20 items forming a single latent factor, five 5-item correlated factors, and five 5-item factors forming one higher-order latent factor. The following four fit indices were used to indicate model-data fit (Hu & Bentler, 1999): the Comparative Fit Index (CFI ≥ .90 for acceptable fit), the Tucker–Lewis Index (TLI ≥ .90 for acceptable fit), the Root-Mean-Square Error of Approximation (RMSEA ≤ .10 for acceptable fit) with a 90% confidence interval, and the Weighted Root-Mean-Square Residual (WRMR ≤ 1 for acceptable fit).

Cronbach’s α and McDonald's ω were used to investigate the internal consistency of the SOCS-O and the SOCS-S. Moreover, corrected item-total correlations (*r*_tot_) were calculated for the SOCS-O and the SOCS-S items to examine how each item contributed to the overall scale. A coefficient lower than .30 indicates that an item is measuring something different from the scale as a whole (DeVellis, 1991). Intraclass correlation coefficients (ICC) were calculated based on a mean-rating (k = 2), absolute-agreement, 2-way mixed-effects model to evaluate the SOCS-O and SOCS-S test-retest reliability. Convergent validity was estimated by calculating Pearson correlation coefficients between the SOCS-O, the SOCS-S, and the remaining measures (SCS-SF, RSE, B-IRI, FCS, SWEMWBS, and BCSQ-12). The strength of the correlations was interpreted following Cohen’s guidelines (Cohen, 1988): small (*r* = .10–.29), medium (*r* = .30–.49), and large (*r* = .50–1.00).

As the primary analysis of the present study, we used path analysis models to explore the mediator role of self-compassion facets. This approach allows for simultaneous estimation of relationships among variables and includes mediation effects. In our investigation, we evaluated a model where self-esteem (RSE) served as the independent variable, the five self-compassion facets (SOCS-S) acted as mediators, and well-being (SWEMWBS) was the outcome. We calculated standardized regression coefficients (β) for bias-corrected bootstrapped indirect effects using 10,000 bootstrap samples, along with their standard errors and 95% confidence intervals (CI). Indirect effects were deemed statistically significant if the 95% CI did not encompass zero (Lockhart et al., 2011), and the model included compassion for others (SOCS-O) as a covariate. The dataset and syntax used for the present study can be found in Pérez-Aranda et al. (2025).

## Results

### Characteristics of the Sample

The survey was completed by 438 participants. However, 30 were excluded for two main reasons: failing to answer a control item designed to detect random responding (*n* = 15) or not being native Italian speakers (*n* = 15). Control items were embedded in some questionnaires to identify inattentive responses, while non-native speakers — mainly international students — were excluded to minimize potential cultural and linguistic bias. Therefore, 408 participants (84.6% women, with a *M*_age_ of 21.07) were included in the analyses. All data provided by the participants were complete; no missing responses were recorded. Most of them (*n* = 249, 61%) were university students of healthcare professions (e.g., medicine, nursing, midwifery), while a notable proportion were in their last year of high school (*n* = 155, 38%). One-third of the sample (*n* = 135) were working and, in terms of income, most considered their financial situation to be around average (see Table 1).

**Table 1:** Baseline Characteristics of the Sample (*n* = 408)

Sociodemographic variables	
Gender: *n* of females (%)	338 (82.8%)
Age: *M*(*SD*)	21.07 (3.54)
Studies: *n* (%)	
High school	155 (38%)
Medicine	134 (32.8%)
Nursing	91 (22.3%)
Midwifery	23 (5.6%)
Resident	3 (0.7%)
Other health care studies	2 (0.5%)
Employment status, n of working (%)	135 (33.1%)
Income: *n* (%)	
Very poor	22 (5.4%)
Below average	134 (32.8%)
Above average	161 (39.5%)
Excellent	58 (14.2%)
Rather not say	33 (8.1%)
Psychological Variable: *M*(*SD*)	
SOCS: Self-compassion [20–100]	69.28 (11.75)
Recognizing suffering [4–20]	15.57 (3.27)
Understanding the universality of suffering [4–20]	17.82 (2.65)
Feeling for the person suffering [4–20]	11.77 (3.18)
Tolerating uncomfortable feelings [4–20]	11.11 (3.09)
Acting to alleviate the suffering [4–20]	13.01 (3.24)
SOCS: Compassion for others [20–100]	82.08 (9.67)
Recognizing suffering [4–20]	15.47 (2.87)
Understanding the universality of suffering [4–20]	18.12 (2.20)
Feeling for the person suffering [4–20]	16.24 (2.53)
Tolerating uncomfortable feelings [4–20]	15.64 (2.40)
Acting to alleviate the suffering [4–20]	16.62 (2.60)
RSE [0–40]	18.48 (6.52)
SWEMWBS [7–35]	24.39 (4.27)
SCS-SF [1–5]	2.77 (0.70)
B-IRI	
Empathetic concern [1–5]	3.99 (0.71)
Perspective taking [1–5]	3.76 (0.78)
Personal distress [1–5]	2.26 (0.74)
FCS	
Towards others [0–4]	1.46 (0.72)
From themselves [0–4]	1.39 (0.84)
BCSQ-12	
Overload [1–7]	3.45 (1.56)
Lack of development [1–7]	3.04 (1.75)
Neglect [1–7]	2.20 (1.18)

*Note*. The square brackets beside each scale contain the range of their scores. Sussex-Oxford Compassion Scale; RSE = Rosenberg Self-Esteem Scale; SWEMWBS = Short Warwick-Edinburgh Mental Well-being Scale; SCS-SF = Self-Compassion Scale-Short Form; B-IRI = Brief Interpersonal Reactivity Index; FCS = Fear of Compassion Scale; BCSQ-12 = Burnout Clinical Subtype Questionnaire-12 item.

### Psychometric Properties of the Italian Version of the SOCS

Based on the fit indices, significance of factor loadings, and previous validation studies, the five-factor hierarchical model was interpreted as best fitting the data both for the SOCS-O (CFI = .99, TLI = .99, RMSEA = .04; WRMR = 0.86) and the SOCS-S (CFI = .97, TLI = .96, RMSEA = .08; WRMR = 1.50). See the Supplementary Materials for more details on the descriptive statistics of the SOCS-O and the SOCS-S items, the standardized factor loadings for the hierarchical five-factor model, and the fit indices for all the tested CFA models.

Internal consistency of the SOCS-O and the SOCS-S factors was adequate, with Cronbach’s α and McDonald’s ω ranging from .67 to .93 (see Supplementary Materials). The *r*_tot_ of the SOCS-O and the SOCS-S items was greater than 0.30 in all cases, suggesting adequate homogeneity. Finally, test-retest reliability after one month was adequate, with ICC coefficients ranging from .73 to .86, which supports the temporal stability of SOCS scores.

Table 2 shows the correlation coefficients between scores on the SOCS-O, SOCS-S, and other constructs. The SOCS-O had significant and large positive correlations with the “empathic concern” and “perspective taking” subscales of the B-IRI, significant and small positive correlations with the SWEMWBS and the RSE, significant and small negative correlations with the “personal distress” subscale of the B-IRI, the FCO/S, and the “neglect” subscale of the BCSQ12; no significant correlations with the SCS-SF and the “lack of development” and “overload” subscales of the BCSQ12 were observed. The SOCS-S had significant and large positive correlations with the SCS-SF and SWEMWBS, significant and moderate negative correlations with the FCS and the “overload” subscale of the BCSQ12, significant and small positive correlations with the RSE and the “empathic concern” and “perspective taking” subscales of the B-IRI, significant and small negative correlations with the “personal distress” subscale of the B-IRI, the FCO, and the “neglect” subscale of the BCSQ12, and no significant correlation with the “lack of development” subscale of the BCSQ12. Pearson’s correlation coefficients among the SOCS subscale scores were also computed (supplementary materials).

**Table 2:** Correlation Coefficients Between Scores on the Sussex-Oxford Compassion Scales and Other Constructs

Variable	SCS-SF	RSE	IRI-EC	IRI-PD	IRI-PT	FCO	FCS	SWEMWBS	BCSQ12-O	BCSQ12-LD	BCSQ12-N
SOCS-O	.07	.16**	.48***	-.14**	.49***	-.14**	-.10*	.23***	.00	.05	-.12*
Recognizing suffering	-.03	.15**	.33***	-.12*	.35***	-.04	-.04	.14**	.09	.11*	-.06
Understanding the universality of suffering	.11*	.05	.21***	-.07	.27***	-.07	-.05	.17***	-.07	-.02	-.14**
Feeling for the person suffering	-.01	.11*	.56***	-.02	.45***	-.17***	-.06	.14**	.03	-.01	-.07
Tolerating uncomfortable feelings	.17***	.17***	.31***	-.22***	.42***	-.15**	-.13**	.26***	-.11*	.04	-.10*
Acting or motivation to act to alleviate suffering	.04	.14**	.42***	-.09	.39***	-.13**	-.10*	.16***	.04	.05	-.12*
SOCS-S	.58***	.28***	.15**	-.25***	.23***	-.21***	-.39***	.58***	-.33***	-.06	-.22***
Recognizing suffering	.16**	.14**	.16**	-.12**	.11*	-.12*	-.21***	.27***	-.12*	.01	-.10*
Understanding the universality of suffering	.20***	.04	.17***	-.08	.27***	-.08	-.10*	.24***	-.13*	.02	-.13*
Feeling for the person suffering	.60***	.28***	.10	-.20***	.15**	-.20***	-.42***	.57***	-.32***	-.04	-.20***
Tolerating uncomfortable feelings	.64***	.30***	.06	-.35***	.18***	-.21***	-.39***	.56***	-.35***	-.12*	-.22***
Acting or motivation to act to alleviate suffering	.57***	.26***	.08	-.20***	.17***	-.17***	-.35***	.54***	-.31***	-.08	-.20***

*Note.* Sussex-Oxford Compassion Scale; RSE = Rosenberg Self-Esteem Scale; SWEMWBS = Short Warwick-Edinburgh Mental Well-being Scale; SCS-SF = Self-Compassion Scale-Short Form; B-IRI = Brief Interpersonal Reactivity Index; FCS = Fear of Compassion Scale; BCSQ-12 = Burnout Clinical Subtype Questionnaire-12 item.

*p < .05. **p < .01. ***p < .001.

### Path Analysis: Self-Compassion Facets as Mediators of the Relationship Between Self-Esteem and Well-Being

**Figure 1:**
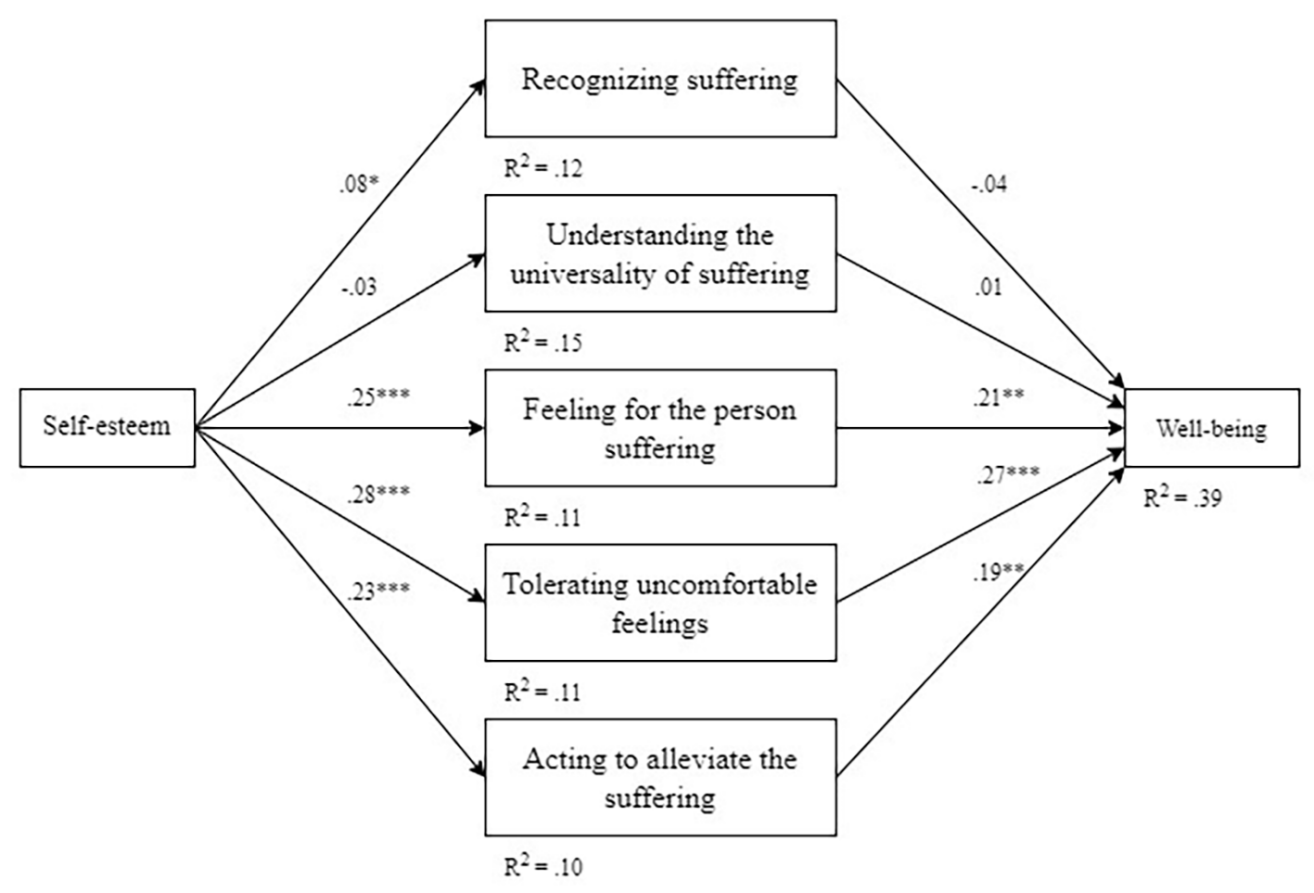
The Final Structure Model With Standardized Path Coefficients *Note. n* = 408. In the model, compassion for others was included as a covariate (β = .09 - .39; *p* < .05 for all cases), and the self-compassion factors were allowed to correlate (*r* = .23–.75; *p* < .001 for all correlations). *p < .05. **p < .01. ***p < .001.

The proposed model adequately fitted with the data (χ12 = 32.54, *p* < .001, CFI = .97, SRMR = 0.026). As depicted in Figure 1, the path analysis model showed that self-esteem significantly predicted recognizing suffering of oneself (β = 0.088, SE = 0.044; *p* = .048), feeling for the suffering of oneself (β = 0.252, SE = 0.050; *p* < .001), tolerating the uncomfortable feelings aroused in response to the suffering (β = 0.277, SE = 0.046; *p* < .001), and acting or being motivated to act to alleviate suffering (β = 0.231, SE = 0.048; *p* < .001). The associations between self-compassion facets were statistically significant (*r* ranging from .26 to .75; *p* < .001 in all cases). Conversely, self-esteem was not a significant predictor of understanding the universality of suffering (β = -0.026, SE = 0.047; *p* = .588).

In turn, well-being was significantly predicted by feeling for the suffering of oneself (β = 0.212, SE = 0.069; *p* = .002), tolerating the uncomfortable feelings aroused in response to the suffering (β = 0.273, SE = 0.059; *p* < .001), and acting or being motivated to act to alleviate suffering (β = 0.190, SE = 0.069; *p* = .006). On the contrary, well-being was not significantly explained by recognizing one’s suffering (β = -0.036, SE = 0.047; *p* = .450) or understanding the universality of suffering (β = 0.007, SE = 0.049; *p* = .883).

Finally, compassion for others (SOCS-O) was included as a covariate of the model, and its effect was significant in every variable (β ranging from 0.091 to 0.392; *p* < .05 in all cases). Overall, the associations proposed in the model explained 39% of the variance of well-being and between 10 and 15% of the variance of self-compassion facets. See R-square estimates in Figure 1.

Regarding the mediating effect of self-compassion facets, the path analysis model observed three significant indirect effects mediating the relationship between self-esteem (RSE) and well-being (SWEMWBS): the self-compassion facets of “feeling for the person’s suffering” (β = 0.054, 95% CI [0.011, 0.097]; *p* = .014), “tolerating uncomfortable feelings” (β = 0.075, 95% CI [0.034, 0.116]; *p* < .001), and “acting to alleviate suffering” (β = 0.044, 95% CI [0.005, 0.083]; p = .029). The remaining self-compassion facets, i.e., “recognizing suffering” (β = -0.003, 95% CI [-0.011, 0.005]; *p* = .430) and “understanding its universality” (β = 0.000, 95% CI [-0.002, 0.002]; *p* = .877), did not show a significant mediating effect.

## Discussion

In line with previous research (Bugay-Sökmez et al., 2023; Holas et al., 2023; Marshall et al., 2015; Pandey et al., 2021, 2023), our study confirms that self-compassion seems to partially mediate the relationship between self-esteem and well-being and our findings suggest that the affective and behavioral facets of self-compassion — which specifically imply emotionally engaging with suffering and making proactive efforts to cope with it — and not the cognitive ones, are responsible of this association. We used the Italian version of the SOCS, which we validated in our sample prior to conducting the mediation analysis. This adaptation demonstrated optimal psychometric properties and maintained the same factorial structure as the original version (Gu et al., 2020) and the Spanish adaptation (García-Campayo et al., 2024).

The results of our path analysis indicate that the self-compassion facets “feeling for the person’s suffering”, “tolerating uncomfortable feelings”, and “acting to alleviate that suffering” constitute significant mediators of the relationship between self-esteem and well-being. These affective and behavioral self-compassion facets are integral to most definitions of compassion (Dalai Lama, 1995; Gilbert, 2009; Neff, 2003a; Pommier, 2010) and require the individual to go beyond mere emotional recognition. Specifically, “feeling for the person’s suffering” is closely related to emotional empathy, i.e., emotionally reacting to what the person is feeling (Shamay-Tsoory, 2011). However, they are not equivalent; while some authors suggest that emotional empathy may precede compassion, others emphasize the distinction between the two constructs, highlighting that compassion involves a caring and supportive attitude toward the person who is suffering, without necessarily vicariously experiencing their distress (Bloom, 2017). On its part, “tolerating uncomfortable feelings” involves abilities such as acceptance, mindfulness, and non-judgement, which are key in “third wave” CBT interventions and have consistently proved their value for increasing well-being (Millard et al., 2023; O’Connor et al., 2018; Wilson et al., 2019). Finally, “acting to alleviate suffering” underscores the necessity of a behavioral response: being self-compassionate implies acting — or, at least, attempting — to alleviate suffering. Our findings suggest that a part of the interaction between self-esteem and well-being is due to the individual’s active efforts to adopt a caring attitude, tolerate discomfort, and try to improve the situation.

Conversely, the cognitive facets of self-compassion did not significantly influence the relationship between self-esteem and well-being. While “recognizing suffering” and “understanding the universality of suffering” have been included — sometimes implicitly — in various definitions of compassion (Dalai Lama, 1995; Feldman & Kuyken, 2011; Pommier, 2010), our findings suggest that individuals with high self-esteem do not necessarily need to consciously acknowledge their distress or frame it as part of a shared human experience to enhance their well-being. The facet of “recognizing suffering” could be seen as functionally similar to cognitive empathy — that is, the ability to identify and understand another’s emotional state (Goetz et al., 2010; Keltner & Kogan, 2018) — but, in this context, addressed to oneself. Although it is an important component of self-compassion and perhaps a necessary first step, “recognizing suffering” may not be sufficient on its own to serve as a mechanism that promotes well-being in individuals with high self-esteem. Similarly, “understanding the universality of suffering” can be seen as a rational exercise aimed at combating feelings of isolation. However, although acknowledging the shared nature of suffering might help individuals feel less alone, it does not appear to directly contribute to the enhancement of well-being through the self-esteem pathway.

By understanding these processes, psychological interventions can be designed to specifically target the different facets of self-compassion that prove to be more relevant for fostering well-being. For example, to focus on the affective facets of self-compassion, techniques such as mindfulness meditation could help individuals recognize their emotional responses to suffering and develop a nonjudgemental sense of empathy toward themselves (Germer & Neff, 2013; Keng et al., 2011). On the behavioral side, interventions should include strategies that encourage compassionate action, such as guiding patients to engage in acts of self-care when they are feeling down. Furthermore, implementing group workshops where participants share their experiences and practice self-compassion exercises can foster a supportive community environment that enhances both affective and behavioral facets of self-compassion.

Despite the non-significant role of the cognitive facets in our model, it is important not to dismiss their potential relevance in other contexts or populations. Previous research has shown that rationally acknowledging suffering as a common human experience is significantly associated with better mental health outcomes, even in samples of students and health professionals (Hall et al., 2013; Shin & Lim, 2019). Moreover, the contribution of specific self-compassion facets may vary across cultures (Montero-Marin et al., 2018); it could be hypothesized that in cultural contexts that emphasize introspection, interdependence, or collectivist identity, the cognitive dimension of recognizing suffering as universal may carry greater emotional resonance and psychological benefit. Future cross-cultural studies are warranted to examine whether affective and behavioral components of self-compassion consistently exert stronger links to well-being, or whether their impact differs depending on sociocultural values surrounding emotional expression, coping styles, and self-perception.

The findings of this study should be considered in light of certain limitations. First, the sample for this study primarily consisted of university students, most of whom were female and enrolled in health-related fields. This sample is likely not representative of the general population, as health professionals — and students in health-related fields — are known to exhibit high levels of self-criticism and low self-valuation (Bogerd et al., 2023; Legassie et al., 2008), as observed in our sample, with participants scoring relatively low in self-compassion and self-esteem. Indeed, the sample was collected using a convenience sampling strategy, which also limits the generalizability of the results. Moreover, the homogeneity of the sample in terms of age, gender, and cultural context limits the generalizability of our findings — even within the population of health sciences students. As a result, the relationship between self-esteem, the facets of self-compassion, and well-being should be further examined in more diverse populations that include broader sociodemographic and cultural variability. Finally, the translation and adaptation of the SOCS could have been strengthened by incorporating additional methodological procedures, as recommended in specialized guidelines (e.g., Sousa & Rojjanasrirat, 2011), such as inter-rater agreement or validation steps.

### Conclusion

Our findings align with previous research that observed a significant mediating or moderating effect of self-compassion on the relationship between self-esteem and well-being. However, by using the SOCS and the distinction between cognitive, affective, and behavioral facets of self-compassion, we suggest that these effects may be particularly associated with the affective and behavioral components of self-compassion. Our results indicate that a significant part of the well-being experienced by individuals with high self-esteem is due to their tendency to emotionally connect with their own suffering, to tolerate the uncomfortable feelings that may arise in response to that connection, and to take action to alleviate that suffering. The implications of these conclusions extend beyond the conceptualization of self-compassion; our findings can be directly applied to the psychotherapeutic context.

## Supplementary Materials

**Table:** 

Type of supplementary materials	Availability/Access
Data	
SOCS Italy datasets.	Pérez-Aranda et al. (2025)
Code	
Code is not available.	—
Material	
Italian and original English versions of the SOCS-O and of the SOCS-S.	Pérez-Aranda et al. (2025)
Goodness-of-Fit indices for the Italian SOCS (*n* = 408).	Pérez-Aranda et al. (2025)
Descriptive statistics of the SOCS-O items and standardized factor loadings for the hierarchical five-factor model.	Pérez-Aranda et al. (2025)
Cronbach’s Alpha, McDonald’s Omega, and Intraclass Correlation Coefficients for the SOCS.	Pérez-Aranda et al. (2025)
Correlation coefficients among scores on the SOCS.	Pérez-Aranda et al. (2025)
Study/Analysis preregistration	
The study was not preregistered.	—
Other	
Syntax - CFA and path analysis.	Pérez-Aranda et al. (2025)

## Data Availability

The datasets used and/or analyzed during the current study are available at Pérez-Aranda et al. (2025).
